# A matrix model describing host–parasitoid population dynamics: The case of *Aphelinus certus* and soybean aphid

**DOI:** 10.1371/journal.pone.0218217

**Published:** 2019-06-13

**Authors:** James Rudolph Miksanek, George E. Heimpel

**Affiliations:** Department of Entomology, University of Minnesota, Saint Paul, Minnesota, United States of America; University of Catania, ITALY

## Abstract

Integrating elements from life tables into population models within a matrix framework has been an underutilized method of describing host–parasitoid population dynamics. This type of modeling is useful in describing demographically-structured populations and in identifying points in the host developmental timeline susceptible to parasitic attack. We apply this approach to investigate the effect of parasitism by the Asian parasitoid *Aphelinus certus* on its host, the soybean aphid (*Aphis glycines*). We present a matrix population model with coupled equations that are analogous to a Nicholson–Bailey model. To parameterize the model, we conducted several bioassays outlining host and parasitoid life history and supplemented these studies with data obtained from the literature. Analysis of the model suggests that, at a parasitism rate of 0.21 d^−1^, *A*. *certus* is capable of maintaining aphid densities below economically damaging levels in 31.0% of simulations. Several parameters—parasitoid lifespan, colonization timeline, host developmental stage, and mean daily temperature—were also shown to markedly influence the overall dynamics of the system. These results suggest that *A*. *certus* might provide a valuable service in agroecosystems by suppressing soybean aphid populations at relatively low levels of parasitism. Our results also support the use of *A*. *certus* within a dynamic action threshold framework in order to maximize the value of biological control in pest management programs.

## Introduction

The ways in which demography, life history, interspecific interactions, and the biotic or abiotic characteristics of a habitat affect the dynamics of consumer–resource interactions may be investigated through simple experiments and ecological models [1]. Host–parasitoid systems are ideal for these studies not only because of their straightforward structure, but because of their application in the biological control of insect pests [2, 3]. Thus, population modeling has often been used to quantify the importance of parasitoids as natural enemies within a broad range of ecological and evolutionary processes [4]. Other approaches, such as life table analyses, also provide valuable insight into the effect of mortality imposed by parasitoids and other natural enemies on host populations [5, 6].

Matrix population models are well adapted to studying structured populations [7–10], although they have seldom been used to describe aspects of host–parasitoid systems. Yet, these life table-based models have been successfully used in a variety of systems to address heterogeneity in populations and in identifying vulnerable aspects of the life history of a species, making them useful not only in theory, but in evaluating the impact of biological control agents in practice as well [3, 11–15]. For example, Lin and Ives [16] constructed a size-classified matrix model for soybean aphid (*Aphis glycines*) and the parasitoid wasp *Aphidius colemani*, showing that parasitoid preference for larger individuals tended to have the greatest impact on host population growth, and Mills [17] utilized a stage-structured matrix to identify the developmental stages of the codling moth (*Cydia pomonella*) most susceptible to increased parasitism pressure in a competitive environment.

We present a coupled stage-classified matrix model for a host–parasitoid system. Our method of coupling two species follows that of the more-familiar Nicholson–Bailey equations, $N_{t+1}=\lambda N_{t}e^{-aP_{t}}$ and $P_{t+1}=N_{t}(1-e^{-aP_{t}})$, in which *N* and *P* are the host and parasitoid population densities, *λ* is the natural rate of increase for the host, and $e^{-aP_{t}}$ is the escape function [18]. We parameterized the matrix model for the soybean aphid–*Aphelinus certus* system through a series of developmental and behavioral bioassays as well as with data from the literature. The purpose of this model—which may be adapted to describe other host–parasitoid systems—is to (1) evaluate the extent to which *A*. *certus* might suppress soybean aphid populations below damaging levels, (2) generate hypotheses related to the potential economic and environmental effects of *A*. *certus* in biological control of soybean aphid, and (3) investigate the dynamics of interacting stage-structured populations.

## Materials and methods

### Study system

Soybean aphid (*Aphis glycines* Matsumura; Hemiptera: Sternorrhyncha: Aphididae) is an important pest of soybean (*Glycine max* (L.) Merrill; Fabaceae) in North America, and often requires treatment with broad-spectrum insecticides that pose risk to an array of non-target organisms [19, 20]. The practice of biological control reduces risk to beneficial species (such as pollinators and natural enemies) as it complements or acts as an alternative to insecticide use [3, 21]. In North America, the biological control services provided by resident enemies offer some protection against soybean aphid (and reduce its overall environmental impact), but damaging outbreaks still occur [19, 22]. The importation and release of exotic specialized parasitoids from the native range of soybean aphid have been attempted unsuccessfully, and various hypotheses for this lack of establishment have been proposed, such as biotic interference [23], intraguild predation [24], and challenges related to overwintering [25, 26] and dispersal [27, 28].

The Asian parasitoid *Aphelinus certus* Yasnosh (Hymenoptera: Chalcidoidea: Aphelinidae) was evaluated for importation and release against soybean aphid. However, *A*. *certus* was determined to be an unsuitable classical biological control agent because it parasitized a broad range of aphid species during tests in quarantine [29, 30]. In or before 2005, *A*. *certus* was accidentally introduced into North America—possibly during a secondary invasion of soybean aphid—and has since spread throughout the north central United States and southeastern Canada [19, 31, 32]. Recent work on *A*. *certus* in Saint Paul, Minnesota, suggests that this parasitoid may be able to maintain soybean aphid populations below the economic threshold of 250 aphids per plant (the pest density at which management practices should be applied) [33], although a different study in the Montérégie area of Québec, Canada, found that *A*. *certus* only decreased peak aphid population densities (and cumulative aphid-days) by 1–7%, possibly due to low early-season parasitism rates [34]. Thus, the overall impact of *A*. *certus* as a biological control agent of soybean aphid remains uncertain.

### The matrix model

Following Caswell [10], a host population vector **n** (the abundance of each developmental stage in the host population) is projected to *t* + 1 (projection interval = 1 d) using a transition and fertility matrix **A** and the probability of escaping parasitism **H**, as well as with a temperature-scaling matrix **C**_SBA_. Similarly, the parasitoid population vector **p** is projected with the transition and fertility matrix **W** and modified by a temperature-scaling matrix **C**_Ac_. The model also includes a carrying capacity *K* for the total host population *N* following Allen [35] and Jensen [36–38], and this formulation includes the identity matrix **I** such that **HAC**_**SBA**_−**I** is analogous to the intrinsic rate of increase. Thus, the model takes the form

$$n_{t+1}=n_{t}+\frac{K-N_{t}}{K}\left(HAC_{SBA}-I\right)n_{t}$$

$$p_{t+1}=WC_{Ac}p_{t}$$ (1)

The matrices **A** and **W** represent the proportion of individuals in stage *j* (columns) surviving or transitioning to stage *i* (rows) from time *t* to *t* + 1. For the host, the survival probabilities (*P*_*i*_), the transition probabilities (*G*_*i*_), and the fertilities (*F*_*i*_) are reduced by parasitism (*g*_*i*_). Eq (2) details the host transition and fertility matrix **A** as well as the probabilities of escaping parasitism expressed in **H**

$$AH=\left[\begin{array}{ccccc} P_{1} & 0 & 0 & 0 & F_{5}\\ G_{1} & P_{2} & 0 & 0 & 0\\ 0 & G_{2} & P_{3} & 0 & 0\\ 0 & 0 & G_{3} & P_{4} & 0\\ 0 & 0 & 0 & G_{4} & P_{5} \end{array}\right]\left[\begin{array}{ccccc} g_{1} & 0 & 0 & 0 & g_{5}\\ g_{1} & g_{2} & 0 & 0 & 0\\ 0 & g & g_{3} & 0 & 0\\ 0 & 0 & g_{3} & g_{4} & 0\\ 0 & 0 & 0 & g_{4} & g_{5} \end{array}\right]$$ (2)

in which *P*_*i*_, *G*_*i*_, and *F*_*i*_ were calculated assuming a postbreeding census birth-pulse, so that the probability of observing an individual of a specific developmental stage is a function of the sampling period. *P*_*i*_ = *l*(*i*)/*l*(*i–* 1), *G*_*i*_ = *l*(*i*)/*l*(*i–* 1), and *F*_5_ = *P*_*i*_*g*_*i*_*m*_*i*_; *l*_*i*_ is the number or proportion of individuals surviving from *i* − 1 to *i*, *m*_*i*_ is per capita reproduction, and *g*_*i*_ is the proportion of hosts escaping parasitism. *g*_*i*_ was based on a type II functional response for parasitoids attacking hosts that was previously applied to *A*. *certus* by Frewin et al. [31] and takes the form

$$g_{i}=\text{exp}\left(\frac{-a_{i}\alpha P_{3}p_{3♀}}{1+a_{i}\alpha T_{h}N}\right)\left(\frac{\sum_{i=1}^{5}n_{i}}{N}\right)$$ (3)

in which *a*_*i*_ is the fraction of all attacks on host stage *i*, *α* is the instantaneous search rate of the parasitoid, and *T*_*h*_ is the handling time. While $\sum_{i=1}^{5}n_{i}$ represents the total number of unparasitized hosts (the scalar sum of the host population vector **n**), *N* represents the entire host population, including both the unparasitized ($\sum_{i=1}^{5}n_{i}$) and parasitized, but still-living, hosts (the element *p*_1_ in the parasitoid population vector **p**). Because only female parasitoids exert parasitism pressure on the host population, the element *p*_3_ from the population vector **p** is multiplied by the proportion of adult parasitoids that are female, and is represented in Eq 3 as *p*_3♀_. As only unparasitized individuals may be parasitized, the escape function is multiplied by the relative number of available hosts, $\sum_{i=1}^{5}n_{i}/N$. Note that the stage-specific probability of escaping parasitism *g*_*i*_ was referred to as “*p*_*i*_” by Lin and Ives [16]; the symbol for this variable was changed here for clarity as entries in our parasitoid population vector **p** are referred to as *p*_*i*_ in conventional matrix notation.

The transition and fertility matrix for the parasitoid (a combined egg and larval stage, mummy/pupal stage, and adult) is

$$W=\left[\begin{array}{ccc} P_{1} & 0 & F_{3}\\ G_{1} & P_{2} & 0\\ 0 & G_{2} & P_{3} \end{array}\right]$$ (4)

in which *P*_*i*_ and *G*_*i*_ are calculated as before, with the exception of *P*_*3*_, which incorporates host-density-dependent survival of adult parasitoids modeled using the Verhulst model of logistic growth (Miksanek JR & Heimpel GE, unpublished). Here, $P_{3}=\left[l(3)/l(3-1)\right]\theta_{1}/[1+{\theta_{2}e}^{\theta_{3}N_{total}}]$, in which *l* is the proportion of parasitoids surviving as before, *θ*_1_ is the maximum mean adult parasitoid lifespan, and *θ*_2_ and *θ*_3_ are shape and growth rate parameters. The fertility of adult parasitoids is $F_{3}={(1-g}_{i})n_{i}/p_{3}$. Finally, the parasitoid survival and transition matrix **W** was additively decomposed to reflect the effects of the host carrying capacity on parasitoid eggs and larvae (as there is an equal probability of the carrying capacity affecting either parasitized or unparasitized hosts), such that the second line of Eq (1) becomes$p_{t+1}={\left[\begin{array}{c} p_{1}\\ 0\\ 0 \end{array}\right]}_{t}+\left(WC_{Ac}-I\right){\left[\begin{array}{c} p_{1}\\ 0\\ 0 \end{array}\right]}_{t}+WC_{Ac}{\left[\begin{array}{c} 0\\ p_{2}\\ p_{3} \end{array}\right]}_{t}$.

Offspring produced by parasitized hosts were added to the element *n*_1_ (number of 1^st^ stadium hosts) in the population vector **n**_*t*+1_ as $\sum_{i=i}^{5}p_{1}J_{i}\frac{n_{i}}{N}$. Here, post-parasitism reproduction is accounted for by multiplying the stage-specific per capita reproduction of parasitized hosts (*J*_*i*_) and the proportional stage structure *n*_*i*_/*N* with the number of still-living parasitized hosts (*p*_1_). This formulation approximates the stage structure of the parasitized host population by equating it to that of the unparasitized population.

A temperature-scaling matrix was implemented for the host (**C**_SBA_) and parasitoid (**C**_Ac_) in order to adjust population growth rates for temperatures outside of those used in laboratory assays. The temperature-scaling matrices take the form

$$C_{\text{SBA}}=\left[\begin{array}{ccccc} {\text{c}}_{P}{}_{1} & 0 & 0 & 0 & {\text{c}}_{5}{}^{4}\\ {\text{c}}_{1} & {\text{c}}_{P}{}_{2} & 0 & 0 & 0\\ 0 & {\text{c}}_{2} & {\text{c}}_{P}{}_{3} & 0 & 0\\ 0 & 0 & {\text{c}}_{3} & {\text{c}}_{P}{}_{4} & 0\\ 0 & 0 & 0 & {\text{c}}_{4} & {\text{c}}_{5} \end{array}\right]C_{\text{Ac}}=\left[\begin{array}{ccc} {\text{c}}_{P}{}_{1} & 0 & {\text{c}}_{3}{}^{4}\\ {\text{c}}_{1} & {\text{c}}_{P}{}_{2} & 0\\ 0 & {\text{c}}_{2} & {\text{c}}_{3} \end{array}\right]$$ (5)

in which c_*i*_ represents a scaling function for the rate of increase and ${c_{P}}_{i}$ is $\left[1-(1-P_{i})c_{i}\right]/P_{i}$. Thus, the term c_*i*_ adjusts sampling probabilities based on the temperature at which laboratory-conducted assays were performed as $\lambda \left(T\right)/\lambda \left(T_{0}\right)$, in which *T*_0_ = 25°C. As *T* → *T*_max_ (the upper temperature threshold for development), individuals have a decreasing probability of being resampled from *t* to *t* + 1 (${c_{P}}_{i}P_{i}<P_{i}$) and an increasing probability of being sampled in the subsequent developmental stage (*c*_*i*_*G*_*i*_ > *G*_*i*_), with ${c_{P}}_{i}P_{i}+c_{i}G_{i}=P_{i}+G_{i}$. Fertilities (*F*_5_ for the host and *F*_3_ for the parasitoid) exhibit the same trend exponentially, with *c*_*i*_^4^ providing the best fit for the matrix approximation of the native function. Our formulation of **C**_SBA_ and **C**_Ac_ was necessary so that the population growth rate can follow temperature-dependent changes in juvenile development and adult survival and fertility. Direct application of the scaling function, e.g. **p**_t+1_ = *λ*(*T*)/*λ*(*T*_0_)**Wp**_*t*_, would yield the correct rate of population growth but only by adding or removing individuals from the population in a biologically unrealistic manner; although our formulation is an approximation, it holds from 5–30°C, which spans the normal range of average historical daily temperatures during the modeling period.

Temperature-dependent development was added for the host following McCornack et al. [39] and for the parasitoid following Frewin et al. [31]. The McCornack et al. [39] model is a modified Logan [40] model that expresses the intrinsic rate of growth, *r*, as a function of temperature, and incorporates the upper development threshold (*T*_max_, the maximum lethal temperature), the range of thermal breakdown (Δ), and a constant *ρ* so that $r\left(T\right)=e^{\rho T}-e^{\left[\rho T_{max}-(T_{max}-T)/\Delta \right]}$. (For reference, the intrinsic rate of growth *r* was related to the natural rate of increase *λ* using the approximation *λ* = *e*^*r*^.) The model used by Frewin et al. [31] was based on an earlier model by Briere et al. [41] (also based on Logan [40]) and estimates the intrinsic rate of growth *r* given an upper temperature threshold (*T*_max_), a lower temperature threshold (*T*_0_), and a constant *a* so that $r\left(T\right)=aT\left(T-T_{0}\right)\sqrt{T_{max}-T}$. These modified Logan [40] models build on improvements made by Lactin et al. [42] and are advantageous in that they decrease the number of necessary parameters while maximizing their biological relevance; Shi et al. [43] has since proposed a similar model based on physiological mechanisms (enzyme kinetics), but the McCornack et al. [39] and Frewin et al. [31] formulations were selected because they were parameterized for the host and parasitoid species used in our study.

### Bioassays

#### Aphid development

Soybean aphids were observed to determine the amount of time required to reach maturity. Reproducing adult aphids from a mixed-aged colony raised at 25 ± 2°C, 16:8 L:D, were transferred to the underside of an excised soybean leaflet. After 1.5 hr, 1^st^-stadium nymphs (n = 31) were transferred with a fine brush to the underside of a fresh excised leaflet from a V1–V2 soybean plant. Leaflets were positioned vertically with the stem placed in 3 cm^3^ of moist, fine sand at the bottom of a 6 dram plastic vial that was ventilated by puncturing pinholes through the cap. Individual aphids were identified to developmental stage at 12 hr intervals until reaching reproductive maturity. Although nymphs and adults are visually and functionally similar, developmental stage can be distinguished by unique differences in antennal segmentation and caudal morphology; antennal segmentation increases from four (1^st^ stadium) to five (2^nd^ stadium) to six (3^rd^ stadium and higher), and the caudum characteristically increases in size before tapering into an elongated teardrop shape at adulthood [44]. Additionally, 4^th^-stadium nymphs often exhibit the eyespots of well-developed embryos that may be seen through their integument. The presence of exuviae and analysis of exuvial antennal segmentation was also used to confirm stage transitions. The entire assay was conducted in a growth chamber at constant 25 ± 2°C, 16:8 L:D.

#### Parasitoid development

Parasitoids were evaluated for their capacity to complete development on each of five apterous stages of soybean aphid (1^st^–4^th^ stadia and adult). Mummies—the darkened exoskeletal remains of recently killed aphids that contain late larval parasitoids or pupae—were collected from laboratory colonies of *A*. *certus* maintained at 23 ± 2°C, 16:8 L:D (first established in August 2011 with field-collected mummies from Saint Paul and Rosemount, Minnesota). Mummies were stored individually in 0.6 mL plastic microcentrifuge tubes supplied with a droplet of honey water (approx. 50 vol%). Each newly emerged female parasitoid (< 24 hr old, n = 59) was paired with a newly emerged male and observed for copulatory behavior; after copulation, the male was discarded and the female left overnight. Each female was randomly assigned a treatment (one of the five host stages), and twenty soybean aphids of representative size and quality for that stage were transferred from a mixed-aged laboratory colony to the underside of a soybean leaflet placed in a plastic vial (as previously described). The aphids were allowed to settle for ten minutes, after which time the parasitoid was introduced into the tube and left to interact with the aphids for 24 hr. The parasitoid was then removed and the aphids were allowed to continue development. Aphids were checked daily for the formation of mummies, which were individually collected in 0.6 mL microcentrifuge tubes and observed at 3–12 hr intervals for the emergence of adult parasitoids. Hind tibia length was measured for a subsample (n = 194) of the emerged offspring as a proxy for size and fitness. The assay was conducted in a growth chamber at 25 ± 2°C, 16:8 L:D. An ANOVA was used to compare the main effects of host stage, sex, and parental identity on parasitoid developmental time as well as hind tibia length. Tukey’s *post hoc* was used to separate means for multiple comparisons. Differences in parasitoid sex ratio in response to different host stages were determined using a linear model with Tukey’s *post hoc*, and the response (proportion male) was weighted based on brood size. These analyses were performed using the agricolae package and base R version 3.4.4 (The R Foundation for Statistical Computing, 2017).

#### Host-stage preference

To determine host-stage preference (defined as the deviation in the proportion of host stages attacked by a single female *A*. *certus* from random chance when all stages present are of equal abundance), *A*. *certus* mummies were collected and mated as before. At the start of the assay, a single female (n = 73) was allowed to exit the microcentrifuge tube onto the underside of a leaflet containing three each of the 1^st^–4^th^ stadia and apterous adult soybean aphids in a plastic vial (as previously described). Only aphids of visually similar quality and of representative size for their stage were used in the assay. Each parasitoid was allowed to interact with aphids for two hours at 25 ± 2°C, which provides sufficient time to locate and parasitize approximately one host (Miksanek JR, personal observation). Immediately after parasitoid exposure, aphids were stored in 70% ethanol and later dissected to recover parasitoid eggs. Host-stage preference was determined using the Friedman rank-sum test (the package stats in base R), which follows a χ^2^ distribution. Parasitoids that did not oviposit during the assay were excluded from the analysis.

#### Post-parasitism reproduction

Aphids were assessed for their reproductive capacity following parasitism. A single 3^rd^, 4^th^, or adult stadium aphid was collected from the laboratory colony and transferred to the underside of a V1 soybean leaflet, which was situated in a plastic vial as previously described. 1^st^ and 2^nd^ stadium aphids were not included because preliminary testing revealed that these stages do not reproduce prior to mummification. A total of 105 vials were assembled, fifteen for each unparasitized (control) 3^rd^, 4^th^, and adult stadium soybean aphid and twenty for each parasitized 3^rd^, 4^th^, and adult stadium aphid. Adult *A*. *certus* (n = 60) were aspirated from a two-week old laboratory colony and placed individually into the appropriate vials. (In the colony, individual wasps had the opportunity to mate, acquire host handling experience, and feed on honeydew or host hemolymph, thus they were considered to be reproductively, behaviorally, and nutritionally prepared for the bioassay.) Each parasitoid was allowed to interact with its aphid for 24 hr, after which the parasitoid was removed. Aphids were observed daily for 8 d for the production of offspring, and nymphs were removed with each observation. The assay was performed in a growth chamber held at 25 ± 2°C, 16:8 L:D.

Reproduction of parasitized and unparasitized adult hosts was analyzed with a cumulative link mixed effects model (CLMM). This approach consists of a multivariate analysis of variance with a logit link function that assesses ordinal response variables while accounting for random factors. The daily number of offspring was the response variable; treatment (parasitized or control), initial host stage (3^rd^, 4^th^, or adult stadium), and number of days since exposure (1–7, a discrete variable) were fixed effects; and individual aphid was included as a random factor to account for repeated measures. An interaction term between treatment and day was included to account for any time-dependent effects of parasitism (i.e. delayed impact on host reproduction). Pairwise comparisons were determined using a *post hoc* test of least square means with a Bonferroni correction. An ANOVA was performed to compare the number of days between molts for parasitized and unparasitized aphids. Aphids that died within the parasitoid exposure period (e.g. due to host feeding or overstinging) were excluded from the analysis, and aphids in the parasitism treatment that did not mummify by the end of the seven-day study period were excluded as well. The CLMM was analyzed using the ordinal package in R, with ten quadrature points used for Gauss-Hermite likelihood approximation. The package emmeans was used as a *post hoc* test for pairwise comparisons of least square means.

### Model analysis

#### Population dynamics

A 90 d period was simulated given a randomly selected initial number of individuals ranging from 0.3–1.82 hosts and 0.15–4.08 parasitoid mummies per plant. These values represent the range of early-season host and parasitoid densities sampled at four sites surveyed during 2017: Hitterdal, MN (47.0°N, 96.2°E), Starbuck, MN (45.6°N, 95.7°E), Appleton, MN (45.3°N, 95.9°E), and Pipestone, MN (44.0°N, 95.9°E) (United States). In order to reflect natural conditions, the initial stage structure for the host was juvenile-biased as colonizing soybean aphid alatae deposit a few offspring per plant without themselves settling [45]; the initial parasitoid population was similarly biased towards younger stages. Colonization timeline followed field observations: aphids were introduced on June 22^nd^ and parasitoids were introduced 20 d later. Simulations were conducted in R and replicated 10000 times.

#### Parasitism and host suppression

Accurate comparisons of field data and ecological models requires clear differentiation of the various methods of measuring parasitism of a host population. *A*. *certus* and other aphid parasitoids are surveyed during their late larval and pupal stages because, at this point, their host has died, leaving behind a mummy (the darkened exoskeletal remains), which are easily sampled in field settings and identifiable to subfamily or genus [46]. However, the relative abundance of mummies—referred to as *mummy fraction*—is not synonymous with other measures of parasitism. To clarify this terminology, we use *parasitism rate* to denote a temporal unit of measurement expressing an absolute or proportional change in the individuals succumbing to parasitism over time [47]. In contrast, *percent* (or *proportion*) *parasitism* is a unitless measure that compares a subset of hosts (the parasitized) to the larger population at some point in time; percent parasitism is thus the *result* of a specific parasitism rate interacting with other competing rates (birth/death, immigration, dispersal, etc.), following van Driesche [48]. Operating under these definitions and following the format of the matrix model, we define *parasitism rate* as $\sum_{i=1}^{5}\left[\left(1-g_{i}\right)n_{i}/N\right]$, *percent parasitism* as *p*_1_/*N* × 100%, and *mummy fraction* as *p*_2_/(*N* + *p*_2_).

#### Sensitivity analyses

The influence of adult parasitoid lifespan, date of parasitoid colonization, host-stage preference, and mean daily temperature was evaluated in ecologically plausible parameter space. The effect of these parameters on host population densities was calculated as a percent difference in maximum host population density with and without the parasitoid present (“peak pest reduction”). The sensitivity analysis for adult parasitoid lifespan (uncoupled from host density) evaluated a mean adult parasitoid survival period of 2–26 d. Parasitoid colonization was evaluated from 2–32 d after host establishment. For host-stage preference, a total of 21 graded preferences were assessed, which ranged from a strong preference for early-stage juveniles (*a*_1–5_ = {0.50, 0.35, 0.10, 0.05, 0.00}), to no overall preference (*a*_*i*_ = 0.20), to a strong preference for adults (*a*_1–5_ = {0.00, 0.05, 0.10, 0.35, 0.50}). The effects of mean daily temperature were assessed over a range of ± 3°C compared to publicly available historical records from the station GHCND:USC00215204 located at (44.4706°N, 95.7908°E) in Marshall, MN. With the exception of the manipulated parameter, all parameters were the same as previously described and simulated using median starting densities for the host and parasitoid populations.

## Results

### Laboratory assays

#### Aphid development

All aphids successfully reached reproductive maturity within seven days. All adults began reproducing within 24 hours of their final molt, and most produced their first offspring within 12 hours; thus, a significant non-reproductive adult stage (referred to as S5 by Lin and Ives [16]) was not noted in our study.

#### Parasitoid development

**Fig 1:**
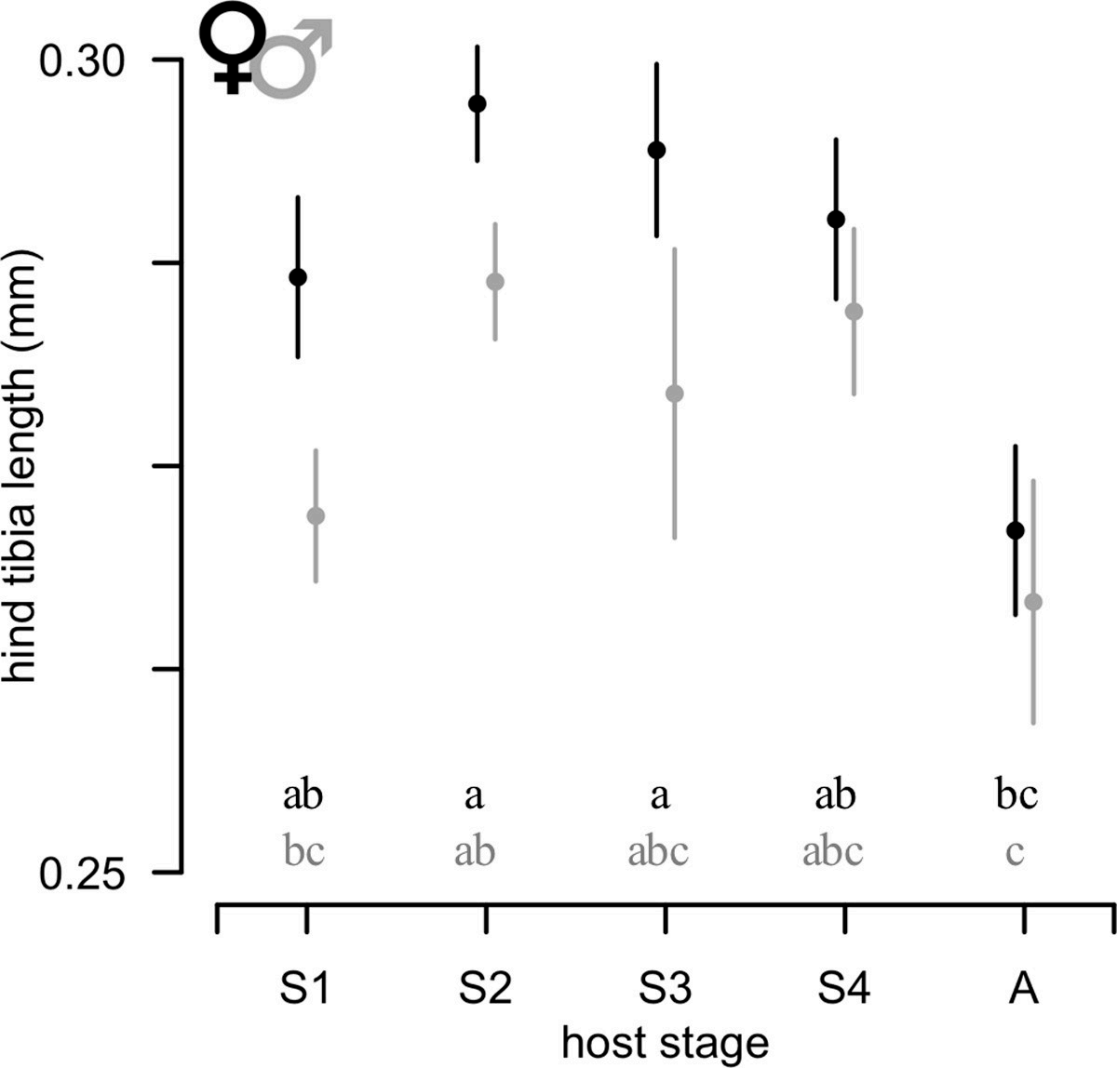
Hind tibia length as a function of host developmental stage. Black: female *A*. *certus*; gray: male. S1: 1^st^ stadium aphid, S2: 2^nd^ stadium, S3: 3^rd^ stadium, S4: 4^th^ stadium, A: adult. Mean ± SE; shared letters indicate no significant difference.

Host stage affected the amount of time required for *A*. *certus* to complete development, both in terms of the time until host mummification (*F*_4, 455_ = 17.23, *p* < 0.001) and time until adult parasitoid emergence (*F*_4, 455_ = 18.87, *p* < 0.001) (Table 1: *Mean time to mummy* and *Mean time to emerge*). Both parasitoid sexes developed more slowly on 1^st^ stadium hosts compared to adult hosts (Table 1: *Total development time*). Males developed more slowly than females (*F*_1, 455_ = 17.38, *p* < 0.001) (Table 1: *Total development time*). The amount of time from mummification to emergence did not differ significantly between sexes (*F*_1, 455_ = 0.06, *p* = 0.807) (Table 1: *Mean time to emerge*). There was an effect of experimental block on developmental rate (time to mummification: *F*_54, 455_ = 6.52, *p* < 0.001; time to emergence: *F*_54, 455_ = 4.89, *p* < 0.001) and size (*F*_52, 136_ = 1.86, *p* = 0.002), indicating similarities among offspring of the same parental parasitoid. Offspring reared on adult hosts were smaller than those developing on other stages (*F*_4,136_ = 8.37, *p* < 0.001), and males tended to be smaller than females (*F*_1, 136_ = 12.66, *p* < 0.001) (Fig 1). A female-biased sex ratio was produced on most host stages; the proportion male was 0.37 ± 0.05, 0.48 ± 004, 0.52 ± 0.04, 0.27 ± 0.6, 0.38 ± 0.6 (mean ± SEM) on 1^st^, 2^nd^, 3^rd^, and 4^th^ stadia and adult hosts, respectively. Although sex ratio varied with host stage (*F*_4, 44_ = 6.49, *p* < 0.001), groups could not be separated *post hoc* by means of Tukey.

**Table 1:** Development time for *Aphelinus certus* on the various stages of soybean aphid separated by parasitoid sex with two-factor comparisons.

	Host-stage	Mean time to mummy (days ± SE) ^a^		Mean time to emerge (days ± SE)^a^		Total development time (days ± SE) ^a^		n
♀	1^st^ stadium	6.18 ± .05	a	7.00 ± .07	a	13.18 ± .07	a	72
	2^nd^ stadium	6.00 ± .05	abc	6.43 ± .08	d	12.43 ± .07	b	67
	3^rd^ stadium	6.02 ± .04	abc	6.60 ± .07	bcd	12.61 ± .09	b	57
	4^th^ stadium	6.08 ± .07	ab	6.47 ± .09	cd	12.55 ± .07	b	66
	adult	5.75 ± .07	de	6.76 ± .07	abc	12.51 ± .09	b	51
♂	1^st^ stadium	6.07 ± .06	abc	6.93 ± .06	ab	13.00 ± .06	a	42
	2^nd^ stadium	5.84 ± .07	cde	6.49 ± .09	cd	12.33 ± .07	b	61
	3^rd^ stadium	5.93 ± .05	bcd	6.46 ± .08	cd	12.39 ± .09	b	56
	4^th^ stadium	5.85 ± .11	bcde	6.65 ± .15	abcd	12.50 ± .07	b	20
	adult	5.57 ± .14	e	6.74 ± .13	abcd	12.30 ± .08	b	23
	*Pooled*:	5.97 ± .02		6.64 ± .03		12.61 ± .03	*Total*:	515

^a^Shared letters indicate no significant difference.

#### Host-stage preference

Parasitoid eggs recovered from dissected aphids were typically located within the anterior abdomen or posterior thorax of the host. Host-stage preference for *A*. *certus* was 0.21, 0.23, 0.21, 0.17, and 0.19 for 1^st^–4^th^ stadia and apterous adults respectively, but did not demonstrate a significant deviation in oviposition from random (Friedman test, *F*_*R*_ = 0.640, *p* = 0.958).

#### Post-parasitism reproduction

**Fig 2:**
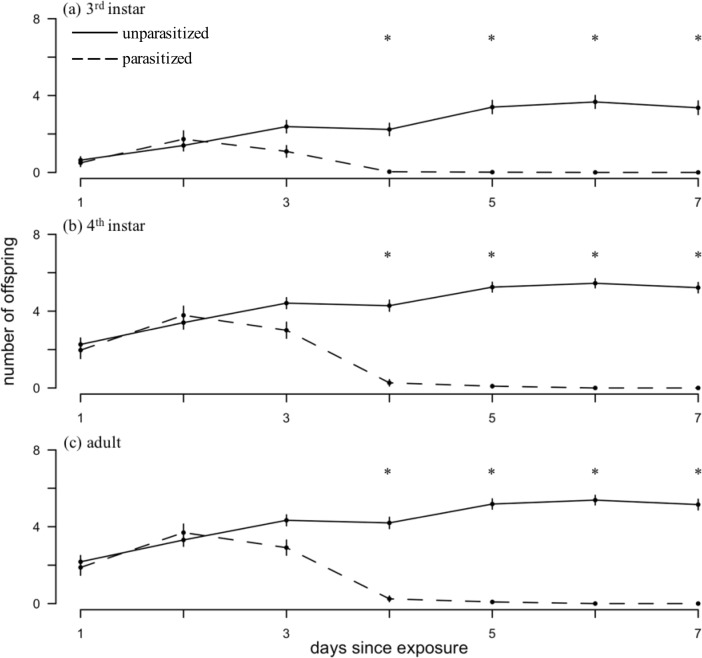
Daily reproduction of parasitized and unparasitized soybean aphids. (a) 3^rd^ stadium, (b) 4^th^ stadium, and (c) adult. Points plot least square means ± SE. Solid lines: unparasitized (control) aphids; dashed lines: parasitized aphids. Asterisks indicate significant differences between control and parasitized aphid reproduction on each day following parasitoid exposure (adjusted for multiple comparisons).

Parasitism by *A*. *certus* negatively affected soybean aphid reproduction (CLMM, likelihood ratio χ^2^_1,440_ = 89.29, *p* < 0.001) and varied by day (χ^2^_6,440_ = 33.91, *p* < 0.001) and with the host stage parasitized (χ^2^_2,440_ = 45.84, *p* < 0.001). Additionally, there was an interaction between treatment and day (χ^2^_6,440_ = 195.44, *p* < 0.001), indicating that the effect of parasitism on host reproduction changed over time (parasitism-induced changes in fertility did not begin until after the third day). The difference in reproduction between parasitized and control aphids was not statistically significant until four days after parasitism, at which time parasitized aphids were rendered infertile (Fig 2). Parasitism did not influence the amount of time between soybean aphid molts (3^rd^ stadium to 4^th^ stadium: *F*_1,20_ = 1.34, *p* = 0.261; 4^th^ stadium to adult: *F*_1,38_ = 0.229, *p* = 0.635).

### Matrix model parameterization

Results from the bioassays were supplemented with data from peer-reviewed literature to parameterize the matrix model. Survival and transition probabilities for soybean aphid nymphs were obtained from the aphid development data, and fertility of parasitized aphids was taken from the post-parasitism reproduction assay. Pooled data from the parasitoid development assay were used determine survival and transition probabilities for immature parasitoids because, even though *A*. *certus* developed more slowly on 1^st^ stadia hosts, this difference was relatively small. Parasitoid sex ratio data were obtained from the parasitoid development assay, and adult parasitoid survival was calculated using unpublished data (Miksanek JR & Heimpel GE, unpublished). Because *A*. *certus* did not exhibit a significant host-stage preference, the null hypothesis *a*_*i*_ = 0.2 was used in the model. The remaining parameters in the model were obtained from the literature, and all parameters used in the model are summarized in Table 2.

**Table 2:** Values and sources of parameters used in the matrix model.

	Parameter	Symbol(s)	Value(s) (excluding units)	Source(s)
soybean aphid	juvenile survival probabilities	*P*_*1*_, *P*_*2*_, *P*_*3*_, *P*_*4*_	0.500, 0.143, 0.311, 0.205	bioassay
	adult survival probability	*P*_*5*_	0.86	[16]
	transition probabilities	*G*_*1*_, *G*_*2*_, *G*_*3*_, *G*_*4*_	0.484, 0.857, 0.689, 0.795^a^	bioassay
	per capita reproduction	*F*_*5*_	2.56	bioassay, [16]
	post-parasitism reproduction	*J*_1_, *J*_2_, *J*_3_, *J*_4_, *J*_5_	0, 0, 0.563, 1.521, 1.471	bioassay
	temperature-curve	*ρ*, *T*_max_, Δ	34.9, 7.1, 0.14	[39]
	carrying capacity	*K*	6000	[49] (lower estimate)
*A*. *certus*	egg+larval survival probability^b^	*P*_*1*_	0.832	bioassay
	pupal survival probability	*P*_*2*_	0.869	bioassay
	adult survival probability	*P*_*3*_	0.932	Miksanek & Heimpel, unpub.
	transition probabilities	*G*_*1*_, *G*_*2*_	0.168, 0.131	bioassay
	sex ratio	–	0.412	bioassay
	host-stage preference	*a*_*i*_	0.2 (H_0_)	bioassay
	functional response	*α*, *T*_*h*_	0.979, 0.045	[31]
	temperature: egg to mummy	*a*, *T*_0_, *T*_max_	1.19 × 10^−4^, 7.8, 35.7	[31]
	temperature: mummy to adult	*a*, *T*_0_, *T*_max_	1.37 × 10^−4^, 11.6, 36.9	[31]
	host-density-dependent survival	θ_1_, θ_2_, θ_3_,	18.6, 13.5, −0.562	Miksanek & Heimpel, unpub.

^a^There is also a 0.016 probability of sampling a first stadium as a third stadium 24 hours later that was included in the model.

^b^Probability of being resampled as an egg or larva at time *t* + 1 assumes no mortality during this period because egg and larva survival were not measured during the assay.

### Model analysis

#### Population dynamics

**Fig 3:**
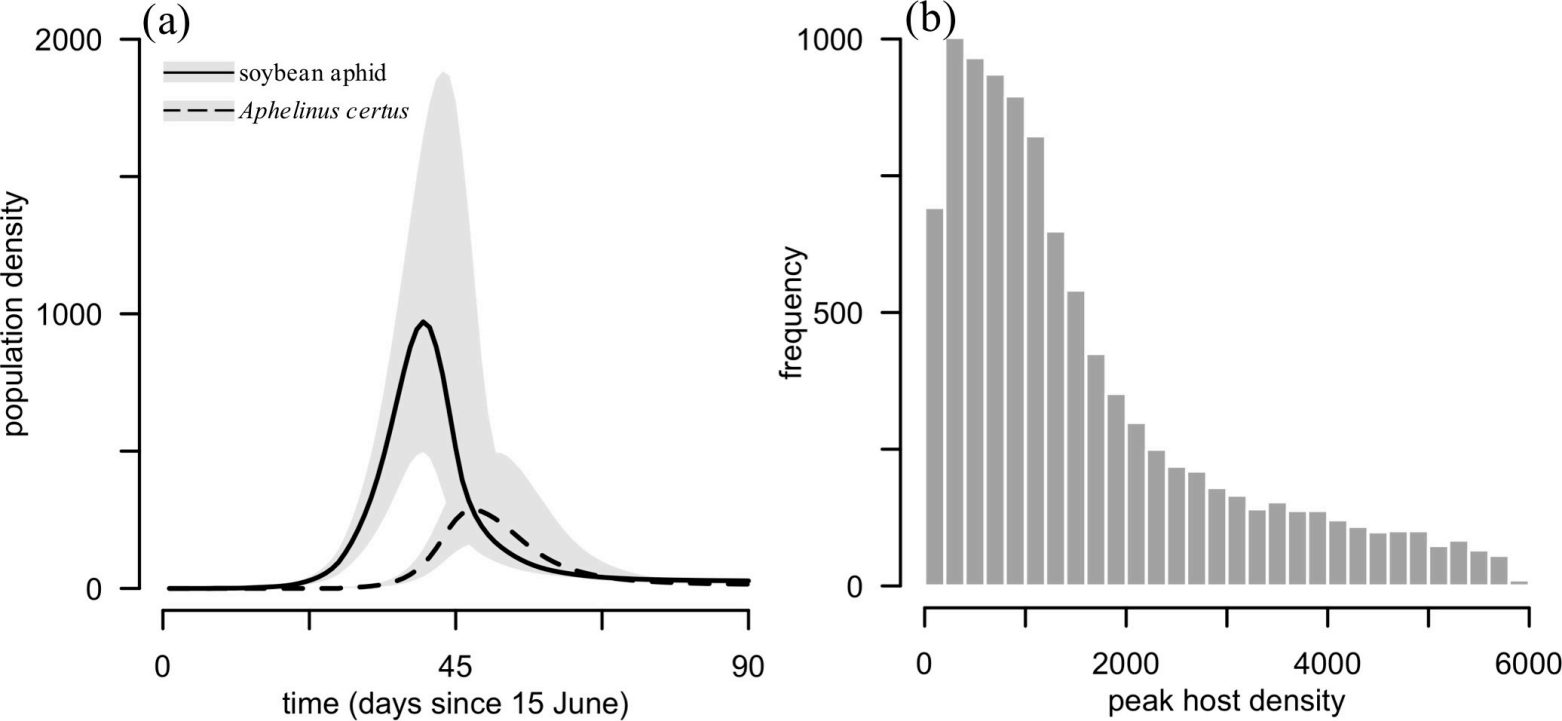
Population dynamics predicted by the matrix model. (a) Population dynamics of soybean aphid and *A*. *certus*. Black lines represent median densities with the interquartile (Q1–Q3) range shaded in gray. Solid line: soybean aphid (all living aphids); Dashed line: *Aphelinus certus* (all stages). Dotted horizontal line: economic threshold. (b) Histogram of peak aphid densities simulated from the model.

Soybean aphid densities peaked just before day 45 of the simulation, which corresponds to the last week of July (Fig 3A). In 9.9% of simulations including *A*. *certus*, soybean aphid densities were below the economic threshold of 250 aphids per plant (the density at which pest management practices should be applied), and in 31.0% did not exceed the economic injury level of 674 aphids per plant (the density at which yield loss exceeds management costs) (Fig 3b). Densities in simulations not including *A*. *certus* reached the carrying capacity of 6000 aphids per plant, and there was a 74.2 ± 0.2% decrease in peak aphid abundance in the presence of *A*. *certus*.

#### Parasitism and host suppression

**Fig 4:**
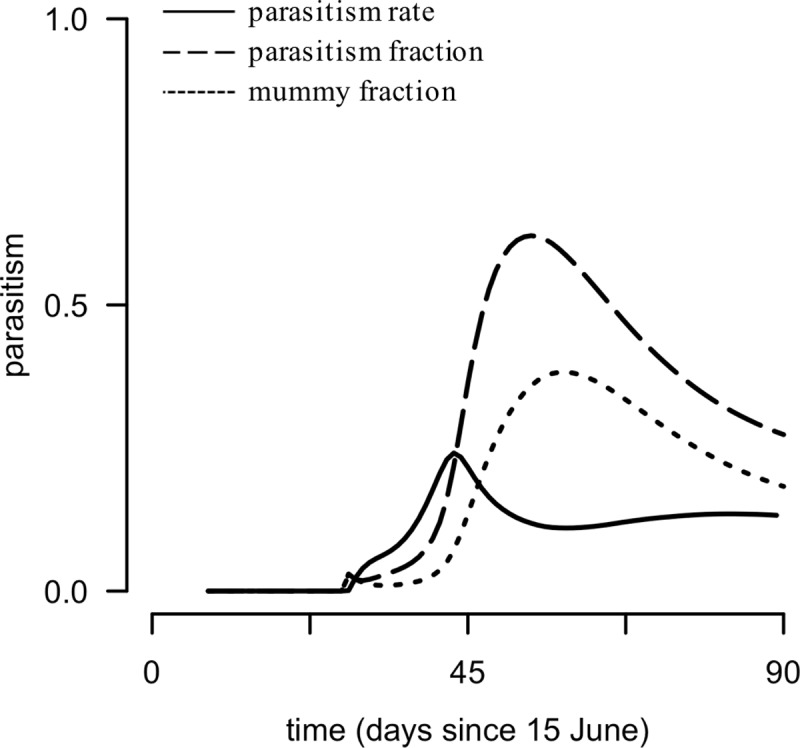
Comparing measures of parasitism in model simulations. Solid line: parasitism rate (d^−1^); dashed line: parasitism fraction (×100 = percent parasitism [%]); dotted line: mummy fraction.

Because the model was stage-structured for both the host and the parasitoid, parasitism rate at any time throughout the season can be equated with mummy fraction and percent parasitism (Fig 4). The parasitism rate associated with no host population growth (*λ* = 1, or the apex of peak aphid density for each of the 10000 simulations) was 0.208 ± 0.012 d^−1^ (mean ± SD), which equates to 11.3 ± 3.7% parasitism or 3.4 ± 1.4% mummies (Fig 4). Regardless of the method of measuring parasitism, parasitism increased with host density before decreasing as the host population declined; however, time-dependent measures of parasitism (percent parasitism and mummy fraction) exhibited a notable lag in comparison with parasitism rate (Fig 4).

#### Sensitivity analyses

**Fig 5:**
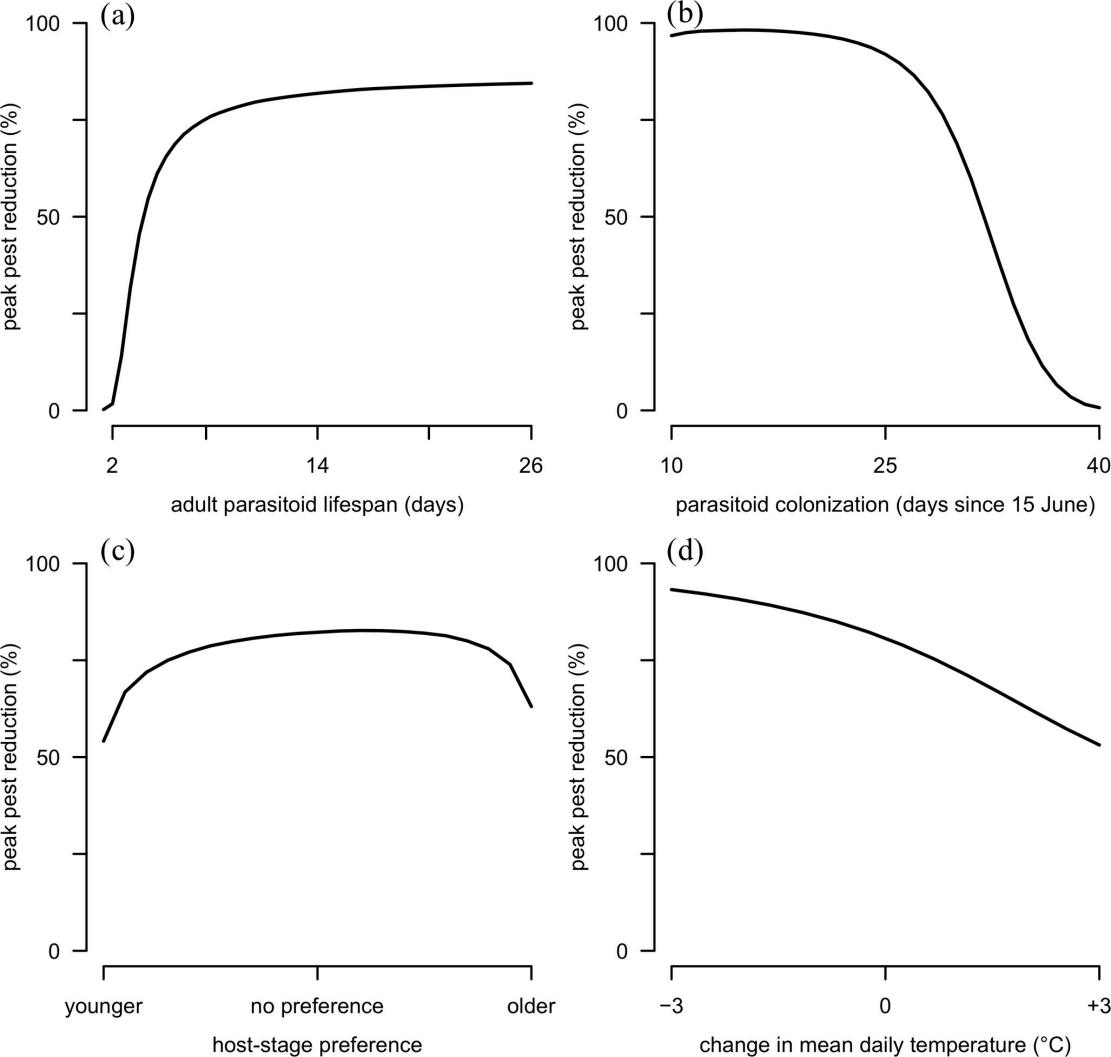
Sensitivity analyses. The effect of the parasitoid is shown as a percent reduction in peak host densities (solid line) as a function of (a) adult parasitoid lifespan, (b) the date of parasitoid colonization, (c) host-stage preference, and (d) mean daily temperature.

Biological control efficacy of *A*. *certus* was greatest for long-lived parasitoids that colonized fields early and attacked hosts without a strong preference for either younger or older host stages. Longer-lived adult parasitoids had a higher impact on the aphid population, and the slope of this relationship was greatest when parasitoid longevity was less than 10 days (Fig 5a). Delaying the date of parasitoid introduction greatly reduced the effect of *A*. *certus* such that, for parasitoids colonizing fields more than a month after the arrival of soybean aphid, their effect was nearly zero (Fig 5B). An increase in parasitoid preference from younger to older hosts produced a concave response in peak pest reduction, indicating that parasitoids attacking all host stages indiscriminately have the greatest effect on aphid population dynamics (Fig 5C). Additionally, lower temperatures were more conducive to host suppression (Fig 5D). Finally, post-parasitism reproduction had a modest effect on peak population reduction; exclusion of this term from the model increased peak population reduction by 0.08% (no figure).

## Discussion

The developmental bioassays revealed a host with a high capacity for growth and a parasitoid that exhibits optimal growth on intermediate host stages but without a significant preference for any individual stage. The fully parameterized matrix model predicted that *Aphelinus certus* reduces soybean aphid populations below the economic threshold in 9.9% of simulations and below the economic injury level in 31.0% of simulations. Host suppression was predicted at a parasitism rate of 0.21 d^−1^, which corresponds with 3.4% of the aphid population being visibly mummified; notably, because parasitism was dynamic, relatively low parasitism rates early in the season could still be associated with low peak host densities. Assuming that the 9.9% modeled reduction in fields exceeding threshold due to parasitism by *A*. *certus* is scalable, then *A*. *certus* might reduce insecticide applications by 1.8 million acres annually, saving $2.43/ha in management costs and contributing to a commensurate reduction in greenhouse gas emissions [22, 50–52]. Our assessment of *A*. *certus* supports the conclusions of Hallett et al. [53] in calculating the value of this parasitoid for implementation in a dynamic action threshold, which would adjust the traditional economic treatment threshold for a pest based on the relative abundance of its natural enemies [54].

### Coupled host–parasitoid dynamics

The matrix model described increasing soybean aphid densities that peaked in late July. This pattern is characteristic of soybean aphid population dynamics in North America [45, 55]. In its native range in Asia, soybean aphid exhibits the same early/mid-season peak, although densities are considerably lower overall and midsummer migrations are of decreased importance [56, 57]. In our model, this unimodal pattern of soybean aphid abundance was driven by a single natural enemy, *A*. *certus*. Soybean aphid is limited by a suite of natural enemies in its native range [57, 58], and, in North America, *Harmonia axyridis*, *Coccinella septempunctata*, *Orius insidiosus* (debatably), and *A*. *certus* have been identified as important predators in certain landscapes [33, 53, 59–64].

Our model suggests that *A*. *certus* is capable of suppressing soybean aphid at a parasitism rate of 0.21 d^−1^ (i.e. parasitizing 21% of the total host population per day). This value is consistent with the 20–30% total daily parasitism range required for soybean aphid population suppression previously determined by Lin and Ives [16], but was relatively low in comparison to the field-estimated 42% parasitism rate proposed by Kaser and Heimpel [33]. This discrepancy may be due to different methods of analysis. The matrix model was analyzed using a non-equilibrium approach, and as a result, our model was able to show that parasitism rate fluctuates dynamically in response to aphid population densities, in which high mid-season parasitism rates followed low early-season parasitism, which contributed to an overall increase in percent parasitism over time. This time difference between increasing parasitism rates associated with host suppression and percent parasitism suggests that it may be difficult to identify the impact of *A*. *certus* in field settings until the pest population is already in decline.

### Insights into host–parasitoid dynamics

The sensitivity analyses demonstrated that adult parasitoid lifespan, date of parasitoid colonization, host-stage preference, mean daily temperature, and post-parasitism reproduction all affect peak host densities to some degree, but the ways in which host-stage preference and post-parasitism reproduction influenced the system merit further discussion. Interestingly, we did not find host suppression to be at a maximum when parasitoids preferentially attacked the oldest host stages and we did not identify post-parasitism reproduction as a mechanism of notably increasing peak population densities, both of which are contrary to the results of Lin and Ives [16].

#### Host-stage preference

The parasitoid *A*. *certus* did not show a significant preference for any individual host developmental stage. Although many *Aphelinus* species readily accept all host stages, there is broad variability in host-stage preference [65–68]. While Lin and Ives [16] showed that preference for older host stages produces the lowest equilibrium host densities, we found that the relationship between host-stage preference and peak host densities produced a different result in our nonequilibrium analysis. When preference for younger hosts became less pronounced, peak aphid densities began decreasing, which is consistent with parasitoids removing hosts of a higher reproductive value [16]. However, as preference for older individuals continued increasing, peak aphid densities began to rise again. In our model, the initial aphid population consists predominately of immature host stages, which mimics the conditions imposed by colonizing alate aphids at the beginning of the season [45]; thus, the adults are much scarcer, so parasitoid preference for late-stage hosts suggests that parasitism rate will be low until their relative abundance eventually increases and the host population approaches its stable stage structure.

#### Post-parasitism reproduction

Soybean aphid reproduction was decreased 72 hr after parasitism by *A*. *certus*, and soybean aphids were reproductively dead the following day. Compared to parasitism by the aphidiine *Aphidius colemani* [16], soybean aphids parasitized by *A*. *certus* reproduce a full day longer, but compared to parasitism by the aphidiine *Binodoxys communis* [69], post-parasitism reproduction was similar. Aphid parasitoids decrease host reproduction when they compete with developing host embryos for nutritional resources [70], which indirectly leads to embryonic degeneration via starvation [71]. Additionally, parasitoids influence the fertility of their hosts by venomous castration [72] or by directly feeding on embryos [71]. In response, parasitized aphids may allocate additional resources to any surviving embryos [69]. Lin and Ives [16] showed that continued reproduction by parasitized aphids during the early stages of parasitoid development produces a partially compensatory effect that leads to higher population growth rates compared with non-reproducing parasitized hosts, and this compensation may be high enough that parasitoids attacking adult aphids—especially older adults—do not affect the maximum growth rate (*r*_m_) or doubling time of their host populations [73, 74]. However, our model did not indicate a strong effect of post-parasitism reproduction on peak soybean aphid densities. Instead, our analysis supports the hypothesis that total lifetime reproduction of aphids has little impact on population growth rates and that the reproductive output during early adulthood contributes disproportionately to population growth (e.g. van Steenis and El-Khawass [75] and references therein). As a result, preference for the oldest host stages allows for high survival for mid- to late-stage immature hosts, which then mature and begin reproducing before succumbing to parasitism themselves.

### An alternative modeling approach

A different approach to modeling herbivorous pest species of annual crops—aphids in particular—involves a linear decline in the intrinsic rate of growth, *r*, due to bottom-up effects of decreasing plant quality as a result of plant phenology; this approach is termed the *decreasing* r *model* [76, 77]. The decreasing *r* model produces a distinctive bell-shaped population curve defined as $N_{t}=N_{0}e^{r_{max}t(1-0.5at)}$, in which *N* is aphid density, *r*_max_ is the maximum rate of population growth, *t* is time, and *a* is the rate of decline for *r*. Decreasing *r* was field-validated for soybean aphid by Costamagna et al. [77] and applied to a host–parasitoid system by Leblanc and Brodeur [34]. Both studies reported a high degree of success using this bottom-up model to describe population dynamics in the field even though soybean aphid dynamics have been previously linked to the strong top-down effect of predation [63].

Decreasing *r* may be incorporated into a matrix model as $n_{t+1}=An_{t}\lambda^{-at}$, in which **n** is the aphid population vector, **A** is the transition and fertility matrix, and *λ* is the dominant eigenvalue of the matrix **A** representing the natural rate of population increase. Analysis of the matrix model (as described in *Materials and Methods*: *The matrix model*) with the addition of decreasing *r* (in which *a* = 0.0247 per Costamagna et al. [77]) predicts that peak soybean aphid densities are reduced by 49.6 ± 0.2% in the presence of *A*. *certus* and, even in the absence of *A*. *certus*, do not exceed the economic injury level, suggesting that soybean aphid might be unlikely to be considered an economically damaging species in any scenario. Regardless, the biggest challenge to the decreasing *r* model in general is that it oversimplifies aphid population dynamics by imposing a season-long effect of plant phenology that confounds density-dependent effects of the aphid on host plant quality with density- and time-dependent changes in aphid behavior and physiology such as emigration, mid-summer migration, or a parthenogenic shift and migration to the primary host plant [78, 79]. Additionally, host population dynamics can be affected by hyperparasitoids and other higher-order natural enemies if, for example, they trigger avoidance behaviors in primary parasitoids or signal hosts of a reduced risk of parasitism, leading to increased reproduction (reviewed by Frago [80]). The overall course of soybean aphid colonization and growth throughout the season is also influenced by landscape-level resource availability, such as proximity to buckthorn or agricultural intensification [53, 81–84].

### Final remarks

Our study highlighted the value of including host stage-specific parameters as well as parasitoid lifespan and colonization timeline in host–parasitoid population models. We also showed a negligible effect of post-parasitism reproduction on peak host densities, and that relatively low parasitism rates early in the season could maintain peak host densities below the economic injury level during the mid-season. Although there have been successes applying real-time monitoring protocols to assess the influence of natural enemies on pest population dynamics and adjust the economic thresholds accordingly (e.g. Hoffmann et al. [85]), such programs can face challenges in development and implementation and are not currently recommended for soybean aphid in the United States [86].
